# NK Cell Priming From Endogenous Homeostatic Signals Is Modulated by CIS

**DOI:** 10.3389/fimmu.2020.00075

**Published:** 2020-01-31

**Authors:** Rebecca B. Delconte, Geoffrey Guittard, Wilford Goh, Soroor Hediyeh-Zadeh, Robert J. Hennessy, Jai Rautela, Melissa J. Davis, Fernando Souza-Fonseca-Guimaraes, Jacques A. Nunès, Nicholas D. Huntington

**Affiliations:** ^1^Division of Molecular Immunology, The Walter and Eliza Hall Institute of Medical Research, Parkville, VIC, Australia; ^2^Department of Medical Biology, Faculty of Medicine, Dentistry and Health Sciences, University of Melbourne, Melbourne, VIC, Australia; ^3^Centre de Recherche en Cancérologie de Marseille, CRCM, Immunity and Cancer Team, Institut Paoli-Calmettes, Inserm, CNRS, Aix Marseille Université, Marseille, France; ^4^Division of Bioinformatics, The Walter and Eliza Hall Institute of Medical Research, Parkville, VIC, Australia; ^5^oNKo-Innate Pty Ltd., Melbourne, VIC, Australia; ^6^Department of Biochemistry and Molecular Biology, Biomedicine Discovery Institute, Monash University, Clayton, VIC, Australia; ^7^University of Queensland Diamantina Institute, Translational Research Institute, University of Queensland, Brisbane, QLD, Australia

**Keywords:** NK cell, CIS, priming, Homeostasis, IL-15

## Abstract

Natural killer (NK) cell activation is controlled by a balance of activating and inhibitory signals and cytokines such as IL-15. We previously identified cytokine-inducible SH2-containing protein (CIS) as a negative regulator of IL-15 signaling in NK cells under inflammatory conditions. While the functional effect of *Cish*-deficiency in NK cells was obvious by their increased anti-tumor immunity and hyper-proliferative response to IL-15, it remained unclear how CIS regulates NK cell biology in steady-state. Here, we investigated the role of CIS in the homeostatic maintenance of NK cells and found CIS-ablation promoted terminal differentiation of NK cells and increased turnover, suggesting that under steady-state conditions, CIS plays a role in maintaining IL-15 driven regulation of NK cells *in vivo*. However, hyper-responsiveness to IL-15 did not manifest in NK cell accumulation, even when the essential NK cell apoptosis mediator, *Bcl2l11* (BIM) was deleted in addition to *Cish*. Instead, loss of CIS conferred a lower activation threshold, evidenced by augmented functionality on a per cell basis both *in vitro* and *in vivo* without prior priming. We conclude that *Cish* regulates IL-15 signaling in NK cells *in vivo*, and through the rewiring of several activation pathways leads to a reduction in activation threshold, decreasing the requirement for priming and improving NK cell anti-tumor function. Furthermore, this study highlights the tight regulation of NK cell homeostasis by several pathways which prevent NK cell accumulation when IL-15 signaling and intrinsic apoptosis are dysregulated.

## Introduction

NK cells are bone marrow (BM) derived lymphocytes that circulate through blood and lymphoid tissues, acting as sentinels of the immune system (1). Their role in protection against cancer has been explored and studies have found increased incidence of cancer in mice and humans that lack NK cells or possess reduced NK cell cytotoxicity (2–4). In addition, a growing number of clinical trials have emerged harnessing the anti-tumor potential of NK cells. It is increasingly evident that NK cell therapies are advantageous over T cell therapies in the clinic in terms of their safety profile (5). Utilizing NK cells in clinical settings such as adoptive cell therapy and chimeric antigen receptor (CAR) engineered cells reduces the incidence of severe cytokine release syndrome (CRS) and graft-versus-host disease (GvHD) side effects (6–8). Improving patient outcomes without contributing to severe side-effects have been the driving force behind improving NK cell immunotherapies and as such, a more comprehensive understanding of the mechanisms regulating NK cell homeostasis have indefinite clinical implications.

We recently identified cytokine-inducible SH2-containing protein (CIS, encoded by *Cish*) as a critical intracellular immune checkpoint in NK cells (9). Indeed, loss of CIS promotes NK cell anti-tumor functions and represents a novel strategy to improve immunotherapy. However, we still do not fully appreciate mechanistically how CIS regulates NK cell anti-tumor functions. We previously showed that CIS regulates IL-15 signaling in NK cells (9). IL-15 is the main driver of NK cell proliferation, survival, differentiation and function, and thus is highly relevant in NK cell homeostasis (10, 11). While the functional effect of *Cish*-deficiency in NK cells was obvious by their increased anti-tumor immunity and hyper-proliferative response to IL-15, it remained unclear how CIS regulated NK cell biology *in vivo* during steady-state.

Whilst NK cells differ from other adaptive immune cells in that they do not require pre-exposure to an antigen to elicit a rapid immune response, there is accumulating evidence implicating the importance of NK cell priming by various cytokines and factors to achieve their maximum response and full repertoire of effector functions. These include IL-15 transpresentation by dendritic cells (12) or macrophages (13), IL-18 (14), IL-12 (15, 16), and Type 1 IFN (17). These data advocate for the notion that there is only a very small fraction of NK cells *in vivo* that qualify as fully-fledged effector cells *in vitro*, and it is likely that these are cells that have been exposed to *in vivo* priming. It would suffice to say then, that NK cell priming can reduce the activation threshold required to elicit a full, directed cytotoxic response toward an infected or cancerous cell. Thus, identifying factors that may reduce this threshold is an important aspect of NK cell biology with the potential to improve NK cell fitness and immunotherapy potential.

To further our understanding of NK cell regulation, we investigated the role of CIS in the homeostatic maintenance of NK cell numbers *in vivo* and evaluated the impact of IL-15 signaling in steady state. In *Cish*-deficient mice (germline, *Cish*^−/−^), the terminal differentiation of NK cells is promoted, as well as increased expression of cell cycle markers. This suggests that even under steady-state conditions, CIS plays a role in maintaining IL-15 driven regulation of NK cells *in vivo*. This homeostatic alteration did not manifest in an accumulation of CIS-deficient NK cells, instead, loss of CIS conferred a lower activation threshold. Global gene expression of *Cish*^−/−^ NK cells compared to WT NK cells revealed upregulation of several signaling pathways and genes important for NK cell function. We conclude that the increased anti-tumor function observed in *Cish*-deficient mice is not caused by homeostatic alterations in, or accumulation of NK cells, but rather the increased anti-tumor effect is a direct consequence of the rewiring of several activation and signaling pathways that results in a reduced activation threshold and decreased requirement for priming.

## Results

### *Cish*-Deficient Mice Have More Terminally Differentiated NK Cells

**Figure 1:**
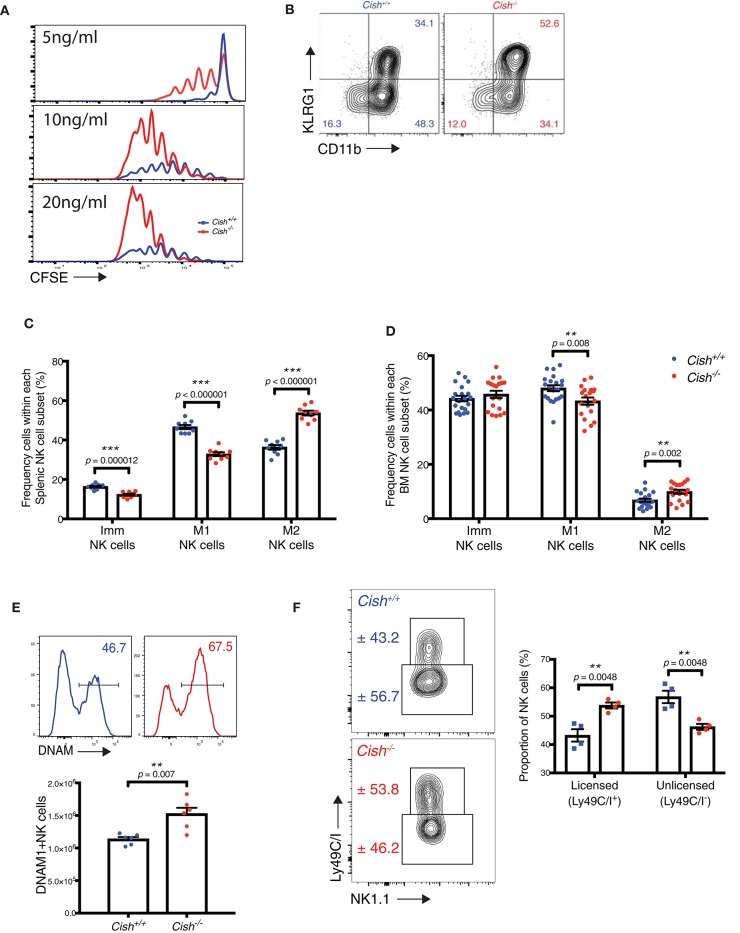
CIS-deficient NK cells show increased maturation. **(A)** Purified and CTV-labeled *Cish*^−/−^ and *Cish*^+/+^ NK cells were seeded at 1 × 10^4^ cells/well into round wells containing different concentrations of IL-15. Cells were incubated at 37°C in a humidified environment containing 5% CO_2_ for 240 h. Representative histogram overlays are shown. **(B–D)** NK cell maturation subsets were quantified by flow cytometric analysis of KLRG1 and CD11b in *Cish*^+/+^ and *Cish*^−/−^ mice. **(B)** Representative FACS plots showing the abundance of each maturation subset and gating strategy in the spleen. Frequency of Imm, M1, and M2 cells were measured within NK cell populations from the **(C)** spleen and **(D)** BM **p < 0.01, ***p < 0.001 (unpaired Student's *t*-test). **(E,F)** Flow cytometry was used to quantify DNAM-1 and Ly49C/I expression. Representative histograms of DNAM-1 expression (**E**, top panel) and Ly49C/I expression (**F**, left panel) and frequency of DNAM-1^+^ NK cells (**E**, bottom panel), and licensed and unlicensed NK cells (**F**, right panel) **p < 0.01 (unpaired Student's *t*-test). (**A,B**, one representative of three experiments; **C**, *n* = 9 biological replicates mean ± s.e.m.; **E**, **F**, values indicate mean ± s.e.m. and *n* ≥ 4 biological replicates).

NK cells with a germline deletion of the *Cish* gene are hyper-responsive to IL-15 due to a lack of receptor signaling dampening (9). Stimulation with both pro-survival and mitogenic concentrations of IL-15 (5 and ≥ 10 ng/ml, respectively) induced enhanced proliferation of *Cish*-deficient NK cells when compared to WT NK cells (Figure 1A). The increased proliferation of *Cish*-deficient NK cells was analogous to increased survival over time, resulting in increased total cell numbers (Supplemental Figure 1A). Despite the robust increase in proliferation and survival *in vitro*, under homeostatic conditions, germline *Cish*^−/−^ mice have normal numbers and frequencies of peripheral NK cells in the blood and all lymphoid organs (Supplemental Figure 1B). We next sought to look at NK cell progenitors in the BM. Previously published RNA sequencing analysis of NK cell progenitors show that NK cell precursors (CD122^+^IL7R^+^NK1.1^−^DX5^−^CD3^−^CD19^−^) in the BM express relatively higher levels of *Cish* than mature NK cells (9). We quantified and compared total cell numbers of NK cell-precursors and observed that they were equivalent and thus unaffected by loss of CIS (Supplemental Figure 1C). In line with previous studies, we also showed that all haematopoietic cells in *Cish*-deficient mice are equivalent in numbers to WT mice (Supplemental Figure 1D).

To explore why *Cish*-deficient NK cells appear “normal” under steady-state conditions, we analyzed the maturation stages of splenic and BM *Cish*^−/^^−^ NK cells. NK cells expressing both NK1.1 and NKp46 mature in a linear fashion from an immature stage CD11b^−^KLRG1^−^ (Imm), through to an intermediate stage (CD11b^+^KLRG1^−^; M1) and finally reach full maturity with the upregulation of KLRG1 [(CD11b^+^KLRG1^+^; M2) Figure 1B] (18). We observed a significant increase in the proportion of mature, M2 NK cells in the spleen of *Cish*^−/^^−^ mice, and this resulted in a compensatory reduction in frequency of M1 and Imm subsets when compared to *Cish*^+/+^ mice (Figure 1C). We also observed a minor increase in the M2 subset in the BM of *Cish*^−/^^−^ mice and a compensatory reduction in the frequency of M1s. However, this was not as dramatic as what was observed in the spleen (Figure 1D). Similarly, gating using conventional markers of NK cell maturation [CD27 and CD11b (19, 20)] also showed an increase in CD27^−^CD11b^+^ mature NK cells and the subsequent decrease in CD27^+^CD11b^+^ intermediates in the spleen and BM (Supplemental Figures 1E,F). These data suggest that the requirement for CIS in controlling NK cell turnover rate and/or maturation is not conserved between the primary site of NK cell generation (BM) and peripheral organs.

To determine whether a loss of CIS manifests in the alteration of other important NK cell receptors in the periphery, we next sought to measure receptor expression on splenic *Cish*^−/−^ NK cells *ex vivo*. We observed a significant increase in the proportion of DNAM-1^+^ NK cells within the *Cish*^−/−^ NK cell population (Figure 1E). The increase in both DNAM-1^+^ and KLRG1^+^ NK cells in *Cish*-deficient mice suggests that under steady state conditions, CIS plays a role in regulating the IL-15-dependent differentiation of NK cells *in vivo*. The preferential increase in DNAM-1^+^ NK cells could also be explained by our previous published data which showed an increase in IL-15 receptor responsiveness of the DNAM-1^+^ proportion of NK cells *in vivo* (21). Furthermore, Ly49C/I receptor expression was altered in *Cish*-deficient NK cells compared to wildtype controls (Figure 1F). Ly49C/I expression defines NK cells into two populations of licensed (Ly49C/I^+^) and unlicensed (Ly49C/I^−^) cells, with each subset possessing differential ability to lyse target cells and respond to infection (22, 23). Studies have shown that licensing is important for NK cells to acquire effector functions. As such, Ly49-deficient mice harbor unlicensed NK cells, which display impaired recognition of MHC-I-deficient target cells and reduced cytotoxicity (24). *Cish*-deficient NK cells show a significant increase in their licensed proportion (Figure 1F), suggesting that they may have a greater cytotoxic capacity and/or augmented activation.

### CIS Governs NK Cell Turnover but Not Accumulation *in vivo*

**Figure 2:**
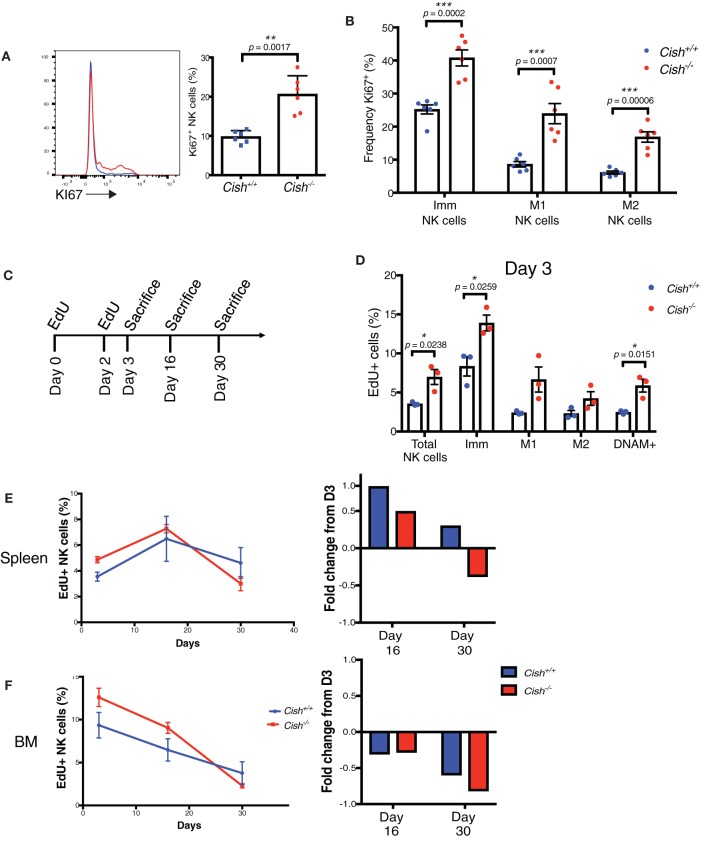
CIS governs NK cell turnover but not accumulation *in vivo*. **(A)** Splenic NK cells from 6 to 8 week-old *Cish*^+/+^ and *Cish*^−/−^ mice were phenotypically analyzed by flow cytometry for Ki67^+^ expression; representative histograms (**A**, left panel) and the percentage of Ki67^+^ NK cells (**A**, right panel) **p < 0.01 (unpaired Student's *t*-test). **(B)** NK cell maturation subsets as shown in (Figure 1B) were analyzed for Ki67 expression ***p < 0.001 (unpaired Student's *t*-test). **(C–F)** NK cell turnover was assessed in 6–8 week-old *Cish*^+/+^ and *Cish*^−/−^ mice using the ClickIT plus EdU staining Kit. **(C)** Schematic showing EdU administration schedule and end points. **(D)** Three days after initial injection, the EdU+ proportion of total splenic NK cells and maturation subsets were analyzed and quantified by flow cytometric analysis *p < 0.05 (unpaired Student's *t*-test). **(E,F)** The percentage of total dividing splenic NK cells and maturation subsets were measured in the spleen and BM (left panels) over time and quantified by flow cytometric analysis. Fold change of EdU uptake or loss over time was calculated using Day 3 as input data (right panels). (**A**, **B**, *n* = 6 biological replicates mean ± s.e.m.; **D–F**, *n* = 3 biological replicates of one experiment representative of two independent experiments with similar results, mean ± s.e.m.).

Published data suggests that KLRG1^+^ NK cells accumulate following periods of acute proliferation (18), thus we next examined whether a loss of CIS induced any changes in proliferation or turnover *in vivo*. Ki67 is a marker associated with cell-proliferation and its expression is absent on resting cells (G0) but is expressed during all active phases of cell cycle (G1, S, G2 and mitosis) (25). *Cish*^−/−^ NK cells show a marked increase in the cell cycle marker, Ki67 in splenic NK cells (Figure 2A). Since there was an observable increase in KLRG1^+^ (M2) cells in *Cish*^−/−^ mice, we sought to understand whether the changes in cell cycle were fixed to one particular subset. Interestingly, Ki67 was increased across all stages of NK cell maturation, including the M2 subset (Figure 2B). To confirm this *in vitro*, the response of each maturation subset to IL-15 was assessed by labeling sorted Imm, M1, and M2 cells with CTV and stimulating them with IL-15 for 5 days. Previous work from our group and others have shown that response to IL-15 signaling is decreased as NK cell maturation increases (18, 20). Intriguingly, while each subset showed an increase in proliferation when compared to their *Cish*^+/+^ counterpart (data not shown), the proliferation profile of the maturation stages was conserved in *Cish*^−/−^ NK cells (Supplemental Figure 2A). Thus, M2 cells were less responsive to IL-15 than M1, and M1 less responsive than Imm. To determine whether IL-15Rβ (CD122) levels could account for the proliferation profile, CD122 expression was also assessed. CD122 expression was found to be increased in the Imm, M1, and M2 subsets of *Cish*^−/−^ NK cells when compared to *Cish*^+/+^ NK cells (Supplemental Figure 2B). However, a loss of sensitivity to IL-15 is still observed as the *Cish*^−/−^ NK cells mature. This is evidenced by the downregulation of CD122 upon KLRG1 upregulation, suggesting that regulation of CD122 is independent of CIS, but dependent on IL-15.

As there was both an accumulation of KLRG1^+^ and Ki67^+^ NK cells in *Cish*-deficient mice, yet no increase in cell numbers, we next asked whether the rate of death of *Cish*-deficient NK cells was increased. The thymidine analog, EdU, is incorporated into DNA during active DNA synthesis and can be used as a measure of cell turnover. Pulse-chase experiments using EdU are commonly used for examining cellular processes over time, thus we assessed the rate of NK cell turnover in the spleen and BM by administering EdU for a set period and subsequently measured the rate of loss of EdU over time (Figure 2C). Consistent with the increase in Ki67, we observed an increase in EdU uptake in total *Cish*-deficient NK cells on Day 3, indicating that CIS is involved in regulating NK cell turnover *in vivo* (Figure 2D). Consistent with the increase in EdU in total NK cells, both the Imm and M1 subsets of *Cish*^−/−^ NK cells showed increased incorporation of EdU, as did the DNAM-1^+^ subset. Previous studies have shown that KLRG1 expressing NK cells are less responsive to proliferative signals *in vivo* (18), and so it was interesting that we observed the M2 subset of *Cish*-deficient NK cells to also show an increase in EdU uptake, albeit not significantly. The increase of KLRG1^+^ NK cells we observed in *Cish*-deficient mice, together with the reduction in Imm and M1 NK cells, and the increased uptake of EdU amongst all three maturation subsets suggests that CIS slows down the rate at which NK cells cycle through from the Imm stage to the M2 stage.

To determine whether the rate of death was increased in the absence of CIS, EdU uptake, and loss were compared over time as outlined in Figure 2C. Measuring EdU uptake over time, we found that the fold change of EdU from initial uptake (at Day 3) was actually increased in the spleen at Day 16, but decreased in the BM. However, when we measured EdU at Day 30, we found that the proportion of EdU^+^ *Cish*^−/−^ NK cells was decreased (Figures 2E,F, left panels). The fold change of EdU^+^ NK cells over time also showed that the loss of EdU from the initial uptake at Day 3 was rapidly lost from *Cish*^−/−^ NK cells in the BM (Figure 2F, right panel). Previous studies have showed that in mice, NK cell turnover in the BM is significantly faster than in the spleen, and that this occurs due to the requirement for precursor populations of NK cells to populate the splenic NK cell niche (26). It is possible that the increase in EdU observed in the spleen at Day 16 is actually EdU^+^ BM NK cells that have migrated to the spleen, and that this must occur continuously during the lifespan of *Cish*-deficient mice due to the rapid turnover of NK cells in the spleen. The increased rate of loss of EdU in Cish^−/−^ NK cells at Day 30 in the spleen is also suggestive of their increased turnover rate and in turn, increased cell death. The rapid cell death did not result in an accumulation of dying cells, ruled out by Caspase 3/7 staining (Supplemental Figure 2C), and similarly there were no significant changes in pro- or anti-apoptotic proteins BIM, MCL1, and BCL2 (Supplemental Figure 2D). Though somewhat surprising, the rapid rate at which *Cish*^−/−^ NK cells are turning over and the subsequent dead/dying cells are cleared by macrophages and phagocytes may explain why there is no observable accumulation. Together, these data suggest that CIS slows down the rate of NK cell turnover and cell death, limiting the responsiveness of NK cells to IL-15 under homeostatic conditions.

### IL-15 Availability Is a Limiting Factor for the Homeostatic Expansion of CIS-Deficient NK Cells *in vivo*

**Figure 3:**
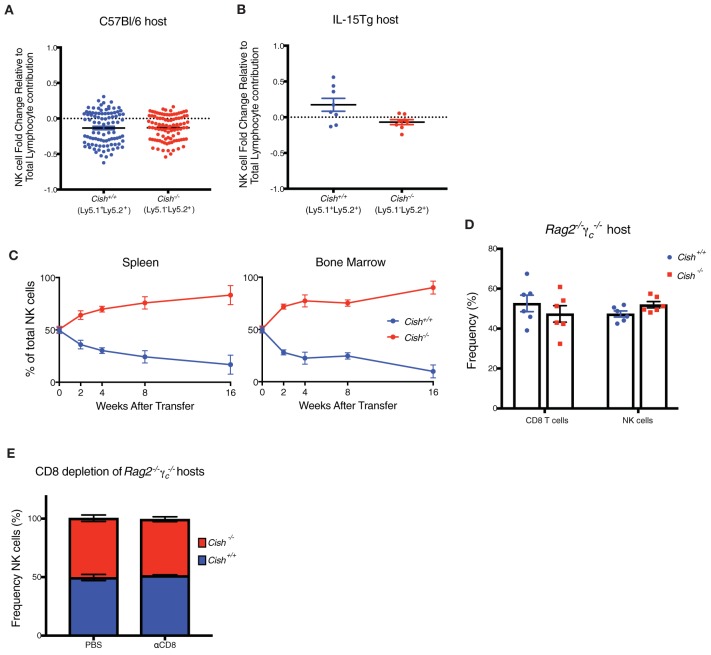
IL-15 availability is a limiting factor for the homeostatic expansion of CIS-Deficient NK Cells *in vivo*. **(A,B)** Haematopoietic chimeras were generated by injecting irradiated host mice (C57Bl/6 or IL-15Tg) with equal amounts of control *Cish*^+/+^ (Ly5.1^+^Ly5.2^+^) and *Cish*^−/−^ (Ly5.2^+^) bone marrow. After 6 weeks, NK1.1^+^NKp46^+^CD49b^+^ cells were analyzed for expression of Ly5.1 and Ly5.2 to deduce donor origin). **(C)** Adoptive transfer models were generated by injecting ${Rag2}^{-/-}\gamma_{c}^{-/-}$ mice with 1 × 10^5^ FACS sorted *Cish*^+/+^ (Ly5.1^+^Ly5.2^+^) and *Cish*^−/−^ (Ly5.2^+^) NK1.1^+^NKp46^+^CD49b^+^ NK cells. NK cells from the spleen (left panel) and BM (right panel) were analyzed for expression of Ly5.1 and Ly5.2 to deduce donor origin at indicated time points. **(D)** Haematopoietic chimeras were generated using irradiated Rag2^−/−^γ_c_^−/−^ mice as hosts and NK and T cells from each donor were assessed as described earlier. **(E)** Reconstituted Rag2^−/−^γ_c_^−/−^ mice were injected i.p. once per week for 4 weeks with anti-CD8 to deplete T cells, and splenic NK cells were analyzed for expression of Ly5.1 and Ly5.2 to deduce donor origin in control (PBS treated) or αCD8 treated reconstituted ${Rag2}^{-/-}\gamma_{c}^{-/-}$ hosts. (**A,B**, *n* ≥ 4 biological replicates mean ± s.e.m.; **C**, mean ± s.e.m. of *n* = 3 biological replicates at each timepoint; **D,E**, *n* = 6 biological replicates mean ± s.e.m.).

Given that *Cish*-deficient NK cells appear to have a heightened sensitivity to IL-15 *in vivo*, we next considered whether this, and the lack of accumulation of NK cells may reflect a consequence of the *Cish-*deficient environment. To do this, we prepared mixed bone marrow (BM) chimeras. Peripheral NK cells from these mice remained at the same proportion as total donor lymphocytes anywhere from 6 to 24 weeks after reconstitution (Figure 3A), and in addition, *Cish*-deficient NK cells maintained an elevated proportion of KLRG1^+^ NK cells (Supplemental Figure 3A). This was despite the increase in the total KLRG1^+^ population of *Cish*^+/+^ NK cells in these chimeras when compared to steady-state, control mice. This suggests there is an increase in turnover rates of NK cells in BM transplant models, which may be caused by the initial total body irradiation (TBI) of the mice, which has shown to produce higher levels of soluble IL-15 and may impact the early stages of NK cell development (27). However, due to the outgrowth of *Cish*^−/−^ NK cells *in vitro*, this suggests the peripheral IL-15 concentration in both TBI and unconditioned *Cish*^+/+^ and *Cish*^−/−^ hosts is below the threshold to stimulate the IL-15 signaling pathway to induce *Cish* expression, thus unable to provide a survival and growth advantage to *Cish* deficient NK cells *in vivo*. To explore this further, we used mice that lack post-transcriptional control of IL-15 gene expression as hosts for mixed BM chimeras (referred to as IL-15Tg). IL-15Tg mice are able to translate and secrete an abundance of murine IL-15 protein, and intact mice display an expansion of NK cell numbers compared to wildtype controls (28). Surprisingly, even in this situation where IL-15 production is substantially increased, peripheral *Cish*-deficient NK cells again remained at the same proportion as total donor lymphocytes and could not outcompete *Cish*-sufficient NK cells (Figure 3B). This was again despite a total increase in KLRG1 expression on both subsets (Supplemental Figure 3B). In a co-culture, *in vitro* setting of IL-15 stimulation, it is apparent that *Cish*-deficient NK cells are hyper-proliferative and superior to *Cish*-sufficient NK cells. Since over-expression of IL-15 *in vivo* could not induce or replicate this competitive advantage, we next questioned whether the presence of IL-15 responsive lymphocytes (other than NK cells) could be causing the homeostatic maintenance of *Cish*-deficient NK cells.

Studies show the lack of NK and CD8 T cells in ${Rag2}^{-/-}\gamma_{\text{c}}^{-/-}$ mice causes the ablation of homeostatic IL-15 sinks, creating an abundance of free soluble IL-15 in the periphery of these mice (29). To address whether the ablation of IL-15 responsive cells (thus an increase in physiological IL-15) could overcome the homeostatic balance between *Cish*^−/−^ and *Cish*^+/+^ NK cells *in vivo, Cish*^−/−^ (Ly5.1^−^Ly5.2^+^) and *Cish*^+/+^ (Ly5.1^+^Ly5.2^+^) NK cells were sorted and adoptively transferred into *Rag2*^−/−^γ_c_^−/−^ mice and their persistence and contribution to the NK cell compartment monitored over 16 weeks. Improved survival of *Cish*^−/−^ NK cells was observed at all time-points and in all analyzed organs post transfer (Figure 3C and Supplemental Figure 3C). The outgrowth of *Cish*-deficient NK cells was accompanied by their sustained increase in the cell-cycle marker, Ki67 compared to WT NK cells (Supplemental Figure 3D). This suggests that the concentration of IL-15 in *Rag2*^−/−^γ_c_^−/−^ mice is high enough to saturate the turnover rate of NK cells *in vivo* and confers a growth and survival advantage to *Cish*-deficient NK cells. While the concentration of soluble IL-15 in TBI mice may be increased at the time of NK cell development in mixed BM chimeras, these data suggest that other γ-chain expressing lymphocytes are still present and acting as homeostatic cytokine sinks, reducing the available IL-15 for NK cells during reconstitution. To determine whether other IL-15 responsive cells are the cause for the lack of expansion of *Cish*-deficient NK cells, we next generated mixed BM chimeras using *Rag2*^−/−^γ_c_^−/−^ mice as hosts. Despite the outgrowth of *Cish*-deficient NK cells when in an adoptive transfer setting, when *Rag2*^−/−^γ_c_^−/−^ mice were used as hosts in a chimeric setting, the competitive advantage of *Cish*^−/−^ NK cells was lost. There was also no competitive advantage given to the *Cish*^−/−^ CD8^+^ T cells (Figure 3D). A previous study showed that depletion of CD8 T cells allowed more room for NK cells to expand, however they also showed that in WT mice that this CD8 depletion alone was not enough to encourage NK cell expansion (30). We hypothesized that the absence of CIS would enhance NK cell sensitivity to the unused IL-15 generated from the absence of CD8 T cells, thus breaking NK cell homeostasis. Mice were depleted of CD8 T cells using a CD8 depleting antibody, and loss of the CD8 T cell population was confirmed by FACS analysis (Supplemental Figure 3E). To our surprise, depletion of CD8s from reconstituted *Rag2*^−/−^γ_c_^−/−^ chimeric hosts did not alter the proportions of *Cish*^+/+^ and *Cish*^−/−^ NK cells from their initial 50/50 reconstitution (Figure 3E), suggesting that IL-15 concentrations were not restored to those in *Rag2*^−/−^γ_c_^−/−^ mice. Similarly, CD8 depletion in intact *Cish*^−/−^ mice did not alter NK cell percentage when compared to WT depletion (Supplemental Figure 3F). The implications from these data are two-fold; (1) CD8 T cells may not be the major IL-15 sink *in vivo* or (2) other γ-chain responsive lymphocytes are responsible for the regulation of NK cell numbers. In either situation, IL-15 availability appears to dictate NK cell expansion, and in steady-state situations there remains alternate regulatory mechanisms in place to maintain NK cell homeostasis.

### Loss of the Pro-apoptotic Protein, BIM, Does Not Alter the Homeostatic Expansion or Anti-tumor Function of *Cish*-Deficient NK Cells

**Figure 4:**
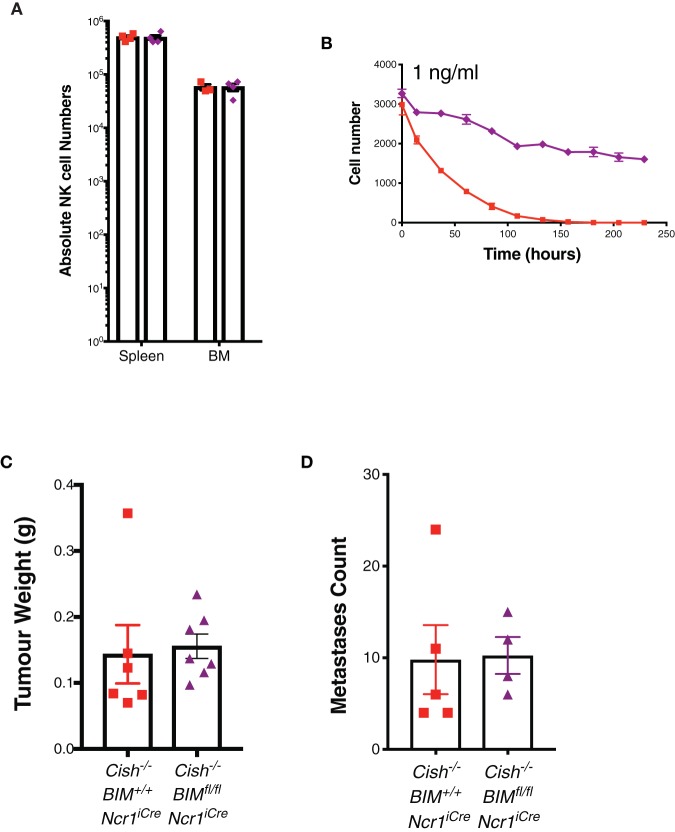
Loss of the pro-apoptotic protein, BIM, does not alter the homeostatic expansion or anti-tumor function of *Cish*-deficient NK cells. **(A)** Absolute numbers of NK cells in the spleen and BM of 6–8 week old female *Cish*^−/−^*Bim*^+/+^*Ncr1*^+/+^*, Cish*^+/+^*Bim*^*fl*/*fl*^*Ncr1*^*KI*/+^*, and Cish*^−/−^ *Bim*^*fl*/*fl*^*Ncr1*^*KI*/+^ mice were quantified by flow cytometric analysis. **(B)** Purified and CTV-labeled *Cish*^−/−^*Bim*^+/+^*Ncr1*^*iCre*^, and *Cish*^−/−^*Bim*^*fl*/*fl*^*Ncr1*^*iCre*^ NK cells were seeded at 1 × 10^4^ cells/well into round wells containing 1ng/ml IL-15. Cells were incubated at 37°C in a humidified environment containing 5% CO_2_ for 240 h. Total cell numbers over time are presented. **(C)** 1 × 10^6^ SM1-LWT1 tumor cells were injected s.c. into flanks of *Cish*^−/−^*Bim*^+/+^*Ncr1*^*iCre*^ and *Cish*^−/−^*Bim*^*fl*/*fl*^*Ncr1*^*iCre*^ mice. After 2 weeks, tumors were excised and volume calculated by weight. (**D)** *Cish*^−/−^*Bim*^+/+^*Ncr1*^*iCre*^ and *Cish*^−/−^*Bim*^*fl*/*fl*^*Ncr1*^*iCre*^ mice were injected i.v. with 5 × 10^5^ SM1-LWT1 cells and 2 weeks later, tumor metastases were enumerated. (**A**, *n* = 4 biological replicates mean ± s.e.m.; **B**, mean ± s.e.m. of *n* = 3 biological replicates at each timepoint; **C,D**, *n* ≥ 4 biological replicates of one experiment representative of two independent experiments with similar results, mean ± s.e.m.).

Previous studies show limiting levels of IL-15 are non-mitotic but are sufficient to maintain NK cell survival by reducing the level of pro-apoptotic protein BIM (encoded by *Bcl2l11*, referred to as *Bim* hereafter) (31, 32). Thus, we next sought to conditionally delete *Bim* in *Cish-*deficient mice using *Ncr1*^*iCre*^ mice to gauge the impact of apoptosis on CIS-null NK cell homeostasis. Surprisingly, *Ncr1*^*iCre*^*Bim*^*fl*/*fl*^*Cish*^−/−^ (referred to as DKO hereafter) mice displayed similar numbers of NK cells to *Ncr1*^*iCre*^*Bim*^+*l*+^*Cish*^−/−^ (referred to as *Cish*^−/−^ hereafter) mice upon phenotypic analysis (Figure 4A). To understand this in more detail, we cultured DKO and *Cish*^−/−^ NK cells in a non-mitogenic concentration of IL-15 and found DKO NK cells appear to survive better under these conditions (Figure 4B). These data suggest that while the pro-apoptotic protein, BIM, may act as an alternative checkpoint in the absence of CIS to ensure the death of rapidly cycling NK cells and maintain homeostatic numbers, in the absence of BIM, *Cish*^−/−^ mice continue to maintain homeostatic NK cell numbers *in vivo*.

There are a number of factors that suppress NK cells in the tumor microenvironment (TME), either directly or indirectly, which can affect NK cell proliferation, effector functions and infiltration [reviewed by (33)]. In the TME, it must be considered that while IL-15 may be upregulated due to the chronic inflammatory nature of tumor formation, other suppressive mechanisms can override robust IL-15 signaling in NK cells under these circumstances. Previous studies have shown *Bim*^−/−^ NK cells can survive IL-15 withdrawal significantly better than WT NK cells and that this is sustained over time (31). This also remains true for DKO NK cells *in vitro*. Thus, we next wanted to look at whether the survival of DKO NK cells could further improve the anti-tumor response of *Cish*^−/−^ mice. The SM1-LWT1 cell line is a metastatic melanoma line and sensitive to NK cell killing (34). We showed in both a sub-cutaneous and i.v. model of SM1-LWT1 that *Cish*-deficient and DKO were similar in their rejection of tumors (Figures 4C,D). Whilst we have previously shown that *Cish-*deficient NK cells are superior at the clearance of tumors, there was no definitive evidence as to what caused this enhanced anti-tumor function. That *Cish*-deficient NK cells still show significant death in low concentrations of IL-15 combined with the similar anti-tumor response observed in DKO mice suggests that increased survival is not a factor contributing to the anti-tumor function of *Cish*-deficient NK cells *in vivo*.

### CIS Regulates Global Gene Expression in NK Cells

**Figure 5:**
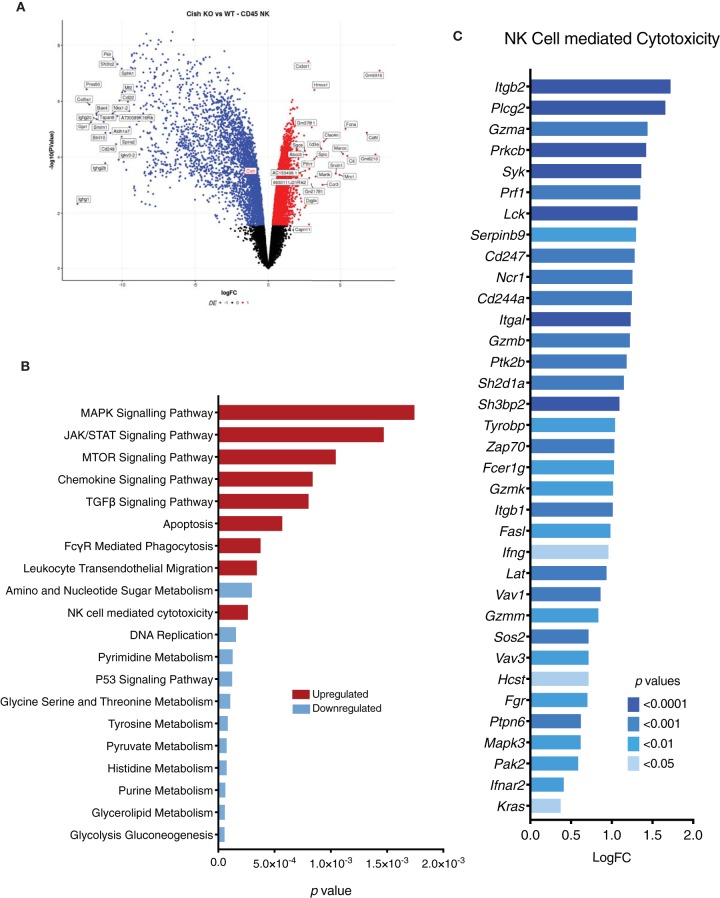
CIS regulates global gene expression in NK cells. Gene expression profiles of sorted NK cells were generated using RNA-sequencing. **(A)** A volcano plot of the top 50 significant (*p* < 0.05) differentially expressed genes with a fold change above +1 or below −1 was generated. y-axis is the negative log-*p*-value and x-axis is the log-fold-change of the corresponding gene in *Cish*^−/−^ vs *Cish*^+/+^ comparison. Gene expression: blue = downregulated, red = upregulated. **(B)** A list of genes was generated using a *p*-value of <0.05 and a cutoff of log(FC) = 1.5. The resulting genes were run through KEGG pathway analysis and 20 of the top enriched KEGG pathways with a *p*-value of <0.01 were plotted. The height of the bar indicates the significance (*p*-value). Pathways: blue = downregulated in *Cish*^−/−^, red = upregulated in *Cish*^−/−^. **(C)** An upregulated list of genes associated with NK cell cytotoxicity (from KEGG pathway analysis) was generated with a *p-*value cut-off of <0.05. x-axis is the log(FC) of each gene and *p*-values are indicated by color.

Since the absence of BIM did not confer a survival or homeostatic advantage to *Cish*-deficient NK cells *in vivo*, we next used RNA sequencing to identify aberrant candidate pathways or genes intrinsic to *Cish*^−/−^ NK cells that may be responsible for regulating their homeostasis *in vivo*. We performed 75-bp single-ended RNA sequencing on *Cish*^+/+^ and *Cish*^−/−^ NK cells purified directly from the spleen. Surprisingly, more than 3,000 differentially expressed genes were detected in *Cish*^−/−^ NK cells. Of these, more than half of the genes expressed at fold-change of 1.5 or higher, and the top 50 DE genes were downregulated, suggesting that a number of active pathways are dampened in *Cish*^−/−^ NK cells in an attempt to counteract the increased turnover and prevent accumulation (Figure 5A). Gene set analysis was indicative of this, with various metabolism pathways downregulated (Figure 5B). From the gene set analysis, it was quite evident the genes that fall within pathways initiated through IL-15 signaling (MAPK, JAK/STAT, mTOR) are upregulated, confirming that *Cish*-deficient NK cells are hypersensitive to IL-15 *in vivo*. However, genes involved downstream of these IL-15 initiated pathways (p53 signaling, DNA replication, and metabolism) are significantly downregulated. This suggests the secondary checkpoints that prevent accumulation are downstream of gene transcription directly initiated by IL-15. Notably, the purine and pyrimidine metabolism pathways were significantly downregulated, along with DNA replication (Figure 5B). In T cells, purine/pyrimidine metabolic processes regulate cell cycle progression and survival, and abrogation of these pathways results in apoptosis (35). The significant downregulation of these pathways in *Cish-*deficient NK cells *in vivo* may help explain inhibitory pathways wired into NK cells to increase apoptosis and prevent accumulation. In addition, a number of genes involved in the apoptosis pathways were upregulated, validating our EdU incorporation results.

Although we observed cyclic abnormalities in *Cish-*deficient NK cells *in vivo*, gene set enrichment analysis showed genes required for NK cell cytotoxicity, FcRγ signaling and chemokine signaling were upregulated. Genes important for activation and degranulation such as *Ifng, Gzma, Gzmb, Prf1*, and *Serpinb9* were upregulated, along with genes downstream of NKG2D and NKp46 activating receptors such as *Hcst, Tyrobp, Zap70*, and *Syk* (Figure 5C). Previously published CHIP-seq data showed that certain STAT5 target genes (such as *Ifng*) are already bound to STAT5 under tonic IL-15 conditions (*in vivo*), and that stimulation with IL-15 increases the amplitude of the gene and number of new binding sites (36). Since tonic levels of IL-15 are similar in *Cish*-deficient and wildtype mice, the increase in STAT5 target gene RNA suggests that *Cish*-deficient NK cells are more sensitive to tonic IL-15 *in vivo*. Increased sensitivity to IL-15 and subsequent enhanced expression of STAT5 target genes such as *Ifng* and *Gzmb* are suggestive of improved activation. As we have seen previously, the absence of CIS confers superior cytotoxicity and subsequent anti-tumor responses. Our data thus far argues that the mechanism behind this is not attributed to tumor infiltration or survival within the tumor microenvironment. Gene set analysis of *Cish*-deficient NK cells shows significant upregulation of pathways and genes associated with NK cell cytotoxicity and activation, and heavily suggests that enhanced anti-tumor function is a direct result of improved cytotoxicity. Interestingly, this is not confined to one singular signaling pathway, but appears to span across a broad range of activating receptors and cytotoxic pathways suggesting that under homeostatic conditions CIS regulates NK cell activation.

### CIS Lowers the Activation Threshold in NK Cells and Increases Anti-tumor Function on a per Cell Basis

**Figure 6:**
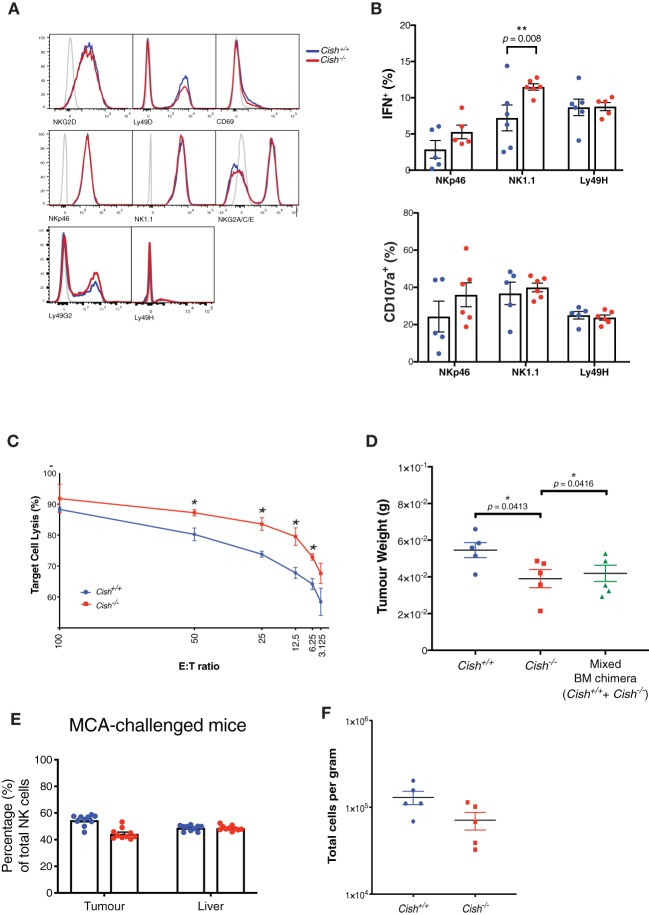
CIS lowers the activation threshold in NK cells and increases anti-tumor function on a per cell basis. **(A)** Splenic NK cells from *Cish*^+/+^ and *Cish*^−/−^ mice were phenotypically analyzed by flow cytometry for activating receptor expression. Representative histograms of receptor expression are shown. **(B)** Total splenic cells were harvested from 6 to 8 week-old *Cish*^+/+^ and *Cish*^−/−^ mice and depleted of red blood cells. Cells were cross-linked with anti-NK1.1, anti-NKp46, and anti-Ly49H antibodies for 4 h in the absence of IL-15. Flow cytometric analysis of NK cells and their IFN-γ production and CD107a (LAMP-1) expression was assessed after 4 h. **p < 0.01 (unpaired Student's *t*-test). **(C)** Splenic NK cells were purified from non-tumor bearing *Cish*^+/+^ and *Cish*^−/−^ mice and cultured with MCA targets at the indicated effector:target (E:T) ratios. Target cell lysis was quantified by detecting the release of a fluorescent cytoplasmic dye into the culture media after 4 h. *p < 0.05 (unpaired Student's *t*-test). **(D)** Female 6–8 week-old reconstituted *Cish*^+/+^, *Cish*^−/−^ and mixed *Cish*^+/+^ and *Cish*^−/−^ BM chimeras were subcutaneously inoculated with 1 × 10^5^ MCA cells. Tumors were excised after 14 days and weighed. *p <0.05 (unpaired Student's *t*-test). **(E)** Tumors and livers from mixed *Cish*^+/+^ and *Cish*^−/−^ BM chimeras were excised 14 days after tumor inoculation and NK cell infiltrate was measured using flow cytometric analysis of Ly5.1 and Ly5.2 to deduce NK cell donor origin. **(F)** Tumors from reconstituted *Cish*^+/+^ and *Cish*^−/−^ mice were analyzed using flow cytometry and total NK numbers enumerated. (**A**, representative plots from *n* = 3 biological replicates; **B**, mean ± s.e.m. of *n* = 3 biological replicates from one experiment representative of three independent experiments with similar results. **C**, mean ± s.e.m. of *n* = 3 biological replicates at each E:T ratio timepoint; **D–F**, mean ± s.e.m of *n* ≥ 5 biological replicates of one experiment representative of two independent experiments with similar results).

The enrichment of genes downstream of activating receptors such as NK1.1, NKp46, and NKG2D suggests a more activated state of *Cish*-deficient NK cells *in vivo*. Thus, we next measured whether there were any alterations in activating receptor expression on the surface of *Cish*^−/−^ and *Cish*^+/+^ NK cells and found no differences (Figure 6A). Our prior studies showed germline deletion of CIS in NK cells manifested in superior IFNγ production when co-stimulated with IL-15 and activating receptors NK1.1 or NKp46 (9). Intriguingly, when *ex-vivo*, germline *Cish*^−/−^ NK cells were stimulated through NK1.1 and NKp46 in the absence of IL-15, we also observed increases in IFNγ production and degranulation compared to *Cish*^+/+^ NK cells (Figure 6B). The functional output from non-specific stimulation (phorbol myristate acetate (PMA) in combination with the calcium ionophore, ionomycin, or IL-12 and IL-18 co-stimulation) was also measured to quantify maximal effector function and interestingly, both IFNγ production and degranulation were equally represented between both genotypes (Supplemental Figure 3G). This suggests that *Cish*-deficient NK cells do not have excess cytokine and degranulation capabilities, but rather, can degranulate and produce cytokine more rapidly from their resting state and suggests that the *in vivo* sensitivity of NK cells to IL-15 establishes a reduced activation threshold. Together with the upregulation of activating genes, it appears that increased sensitivity to IL-15 *in vivo* results in NK cells being “pre-primed” or reducing their activation threshold and that any increases in *Cish*^−/−^ NK cell activation is not an effect of receptor abundance or intensity, but rather downstream effects of the activating receptors.

To evaluate this pre-primed phenotype *in vivo*, we first assessed the differences in cytotoxic capacity between *Cish*-deficient and WT NK cells against an immunogenic methylcholanthrene (MCA)-induced fibrosarcoma cell line (MCA1956). We found that when challenged *in vitro, Cish*^−/−^ NK cells were more efficient at lysing MCA tumor cells at a range of effector:target (E:T) cell ratios (Figure 6C). In *vivo*, 100% *Cish*^−/−^, 100% *Cish*^+/+^ or 50/50 competitive chimeras were challenged with the MCA1956 cell line, and tumor burden was decreased in both the *Cish*-deficient chimeras as well as the competitive chimeras (Figure 6D). When intratumoural NK cell proportions were examined within the 50/50 (*Cish*^−/−^/*Cish*^+/+^) competitive chimeras, in line with our previous reconstitution data from the BM and spleen, the NK cell proportions within the tumor and in peripheral organs remained at 1:1 (Figure 6E). Additionally, in 100% chimeras, despite the decrease in tumor volume, the absolute number of *Cish*^−/−^ NK cells per gram of tumor was not increased compared to *Cish*^+/+^ NK cells (Figure 6F). Together with our previous data that showed in the absence of BIM, NK cell anti-tumor function does not appear to benefit from increased survival, these data suggest that the failure to outcompete WT NK cells in the tumor setting does not compromise the anti-tumor function of *Cish*-deficient NK cells and highlight their increased cytotoxicity on a per cell basis. Thus, the upregulation of cytotoxicity and activating receptor genes in the absence of CIS enhance the effector capabilities of NK cells.

## Discussion

NK cell homeostasis is a tightly controlled event that encompasses a number of complex processes and signaling events. IL-15 is the crucial factor inducing NK cell proliferation, survival, and differentiation *in vivo* (37, 38). The present data identifies a role for CIS in murine NK cell homeostasis and suggests a mechanism for the enhanced anti-metastatic function previously published in *Cish*-deficient mice (9). Whilst tonic levels of IL-15 were initially thought to render the role of CIS redundant under homeostatic conditions, *Cish*-deficient NK cells are terminally mature, show enhanced cycling (Ki67 expression, EdU uptake) and turnover (loss of Edu), arguing in favor of a physiological role for CIS *in vivo*. However, additional safety mechanisms or checkpoints appear to be in place to prevent NK cell accumulation in the absence of this potent negative-regulator of IL-15 receptor signaling.

Alterations in NK cell numbers during health and disease are rarely observed in mice or humans. An exception being the large transient increase in NK cell numbers during murine or human cytomegalovirus (CMV) infection which returns to baseline 2 weeks post-infection and is driven by a viral peptide, not IL-15 (39, 40). NK cell numbers in the periphery of mice lacking genes important for NK cell survival, proliferation, differentiation, or function show very little change in NK cell numbers. In mice where the loss of a gene can dramatically alter NK cell biology *in vitro*, NK cell numbers *in vivo* are not altered. *Cish*-deficient mice (improved IL-15-dependent survival/proliferation of NK cells) and *Bcl2l11*-deficient mice [whereby *Bim* deficiency in NK cells results in a dramatic survival advantage in the absence of IL-15 (31)] are examples of this as both of these models harbor NK cell frequencies equal to that of littermate controls under homeostatic conditions. This is in vast contrast to similar genetic alterations in T and B cells, which result in their accumulation over time leading to immunopathology and autoimmunity (41, 42).

In humans, this tight regulation of NK cells is conserved, evidenced by the rarity of lymphoproliferative disorders of NK cell origin, which account for less than 5% of all lymphoid neoplasms (43). Perhaps even more interesting, is that in the rare cases where NK cell numbers become dysregulated, such as aggressive NK cell leukemia (ANKCL), the prognosis is extremely poor, with a mean survival rate of 2-3 months after diagnosis (44). This in itself may be evidence for why NK cell numbers have a number of safeguards or checkpoints in place when it comes to their regulation *in vivo*. Surprisingly, removing *Bim*-dependent intrinsic apoptosis from NK cells that are hyper-responsive to IL-15 (*Ncr1*^*iCre*^*Bim*^*fl*/*fl*^*Cish*^−/−^*)* did not result in an accumulation of NK cells *in vivo*. It is possible, that alternate apoptotic regulators such as *Noxa* and *Puma* are also upregulated in *Cish*^−/−^ NK cells, and that this can compensate for a loss of *Bim*.

In an attempt to understand how CIS regulates essential NK cell functions, we were able to demonstrate that NK cell increased anti-tumor efficacy in the absence of CIS is not due to NK cell accumulation, increased survival or infiltration at the tumor site. However, our gene set enrichment analysis (GSEA) shows upregulation of signaling pathways essential for NK cell cytotoxicity. This was accompanied by increases in IFNγ production and CD107a expression upon activating receptor stimulation *in vitro*.

The regulation of NK cell responses to infectious or tumor cells is regulated by the balance between activating and inhibitory signals [summarized by (45)]. This implies the existence of an activation threshold, where a certain level of activation must outweigh the inhibitory signals in order to elicit a functional response (46). This study suggests CIS maintains an activation threshold, and that by inhibiting CIS, increased sensitivity to IL-15 is generated and lowers the activation threshold to elicit a functional response. The absence of exogenous IL-15 during the 4 h stimulation assays ensures no induction of CIS, thus it is likely the levels of IL-15 *in vivo* are responsible for the pre-primed response of *Cish*-deficient NK cells compared to WT.

Additionally, it was showed in the early 1980s that a proportion of NK cells are capable of killing multiple targets in succession (47). Additionally, recent reports suggest that IL-15 can improve the killing frequency of NK cells by augmenting the number of targets an NK cell can kill before they become exhausted (48). This study shows *Cish*-deficient NK cells have the ability to lyse more target cells than WT NK cells both *in vitro* and *in vivo* and suggests that CIS regulates, at least in part, the killing frequency of NK cells.

How CIS imposes restrictions on the number of targets an NK cell can kill may occur in a number of ways. *Cish*-deficient NK cells show differential upregulated expression of the gene *Serpinb9*. *Serpinb9* prevents cell death by inactivating proteases such as granzyme B that are released into the host cell interior (49). This suggests a mechanism by which *Cish*-deficient NK cells harbor an increased killing frequency when they encounter tumor metastases *in vivo*. More studies are required to understand the precise mechanisms behind these initial observations, but it suggests that CIS is an early inhibiting molecule of several signaling pathways that regulate NK cell activation thresholds.

In conclusion, targeting CIS improves NK cell *ex-vivo* proliferation, but also demonstrates no homeostatic or developmental differences or abnormal accumulation when CIS is deleted *in vivo*. These results emphasize the safety of targeting CIS, promoting improved anti-tumor functions without any apparent secondary or off-target effects. Importantly, we also highlight the targeted deletion of CIS as a means of lowering the NK cell activation threshold, in turn enhancing anti-tumor function using standard methods of stimulation. Thus, in this study we confirmed and further characterized the inhibition of CIS as a method of unleashing the NK cell anti-tumor response. We propose that releasing NK cell inhibition by targeting CIS is a novel strategy to improve NK cell anti-tumor properties and recommend their use in clinical immunotherapy and adoptive transfer approaches.

## Methods

### Mice

*Cish*^−/−^ were generously provided by J. Ihle and E. Parganas (St. Jude Children's Research Hospital) and were maintained on a C57BL/6 background. *Cish*^+/+^ refers to C57BL/6 wild-type control mice. Age and sex matched mice were used and cohort size was dictated by previous experience using these tumor models. Mice were bred and maintained under specific pathogen-free conditions at The Walter and Eliza Hall Institute. Animal experiments followed the National Health and Medical Research Council (NHMRC) Code of Practice for the Care and Use of Animals for Scientific Purposes guidelines and were approved by the Walter and Eliza Hall Institute Animal Ethics Committee.

### Genotyping

*Bcl2l11*^*fl*/*fl*^ genotyping was performed using the following PCR primers: Bim9, 5′-GACAAGGTGGACAATTGCAG-3′; PB173, 5′-AACCAACTGTACCTTGGCTATA-3′; with expected band sizes at 620 bp for WT alleles and 920 bp for floxed allele. *Ncr1*^*iCre*/+^ genotyping was performed using the following PCR primers: iCRE Fwd, 5′-GGAACTGAAGGCAACTCCTG-3′; iCRE Fwd KI, 5′-GTCCATCCCTGAAATCATGC-3′; Rev WT:-5′ TTCCCGGCAACATAAAATAAA-3′ with expected bands sizes at 300 bp for WT allele and 376 bp for KI allele.

### Generation of BM Chimeras

All BM chimeras were generated using the same method. Host mice were lethally irradiated (2 × 550 rads) and reconstituted with either 7 × 10^6^ donor BM cells for 100% chimeras or 3.5 × 10^6^ control donor BM and 3.5 × 10^6^ experimental donor BM. Mice were kept on neomycin-treated water for 3 weeks, and reconstitution of BM compartment was monitored from 6 weeks onwards.

### NK Cell Cytotoxicity Assays

Standard 4 h cytotoxicity assays were completed as described elsewhere (50). Briefly, splenic NK cells were isolated and suspended in NK cell medium (phenol-red free RPMI 1640 containing 10% FCS, non-essential amino acids, L glutamine and sodium pyruvate, all from Gibco). The indicated target cells were labeled with 15 μM Calcein-AM (Life Technologies) for 30 min at 37°C, washed twice and suspended in NK cell medium. Effector and target cells were combined at the indicated ratios in triplicate wells of a round-bottom 96 well plate and incubated at 37°C/5% CO_2_ for 4 h. Calcein release was quantified by transferring 100 μL of cell-free supernatant to opaque 96 well plates and measuring fluorescent emission at the appropriate wave-length (excitation filter: 485 ± 9 nm; cutoff: 515 nm; emission: 525 ± 15 nm) using the EnVision Robot Plate Reader.

### Flow Cytometry and Cell Sorting

Single-cell suspensions were stained with the appropriate monoclonal antibody in PBS containing 2% FCS. When necessary, intracellular staining was performed by use of the FoxP3/Transcription Factor Staining Buffer Set (eBioscience) according to the manufacturer's instructions. Fortessa, FACS Verse, and ARIAIII (BD Biosciences) were used for cell sorting and analysis. Antibodies specific for NK1.1 (PK136; 1:100), CD19 (1D3; 1:400), CD3 (17A2; 1:400 or REA641; Miltenyi Biotec; 1:150); CD122 (TM-β1; 1:200), NKp46 (29A1.4; 1:100), KLRG1 (2F1; 1:200), CD27 (SB/199; 1:200), CD11b (M1/70; 1:200), IL-7R (A7R34; eBioscience; 1:200) CD49b (DX5; 1:100), CD49a (Ha31/8; 1:200) Ly49H (3D10; 1:200) Ly49D (4E5; 1:200), NKG2D (C4; 1:200), NKG2A/C/E (20d5; 1:200), Ly49C/I (5e6; 1:100), CD107a (104B; 1:100), and IFN-γ (XMG1.2; 1:100), DNAM-1 (10E5; 1:200); Ki-67 (AF488; 1:50) were from BD Pharmingen unless stated otherwise.

### Cell Counts

123count eBeads (BD Bioscience) beads were added to single cell suspensions prior to flow cytometry. Cell numbers were enumerated according to manufacturers instructions.

### Enumeration of Apoptotic Cells

The enumeration of apoptotic cells was performed using the CellEvent^TM^ Caspase-3/7 Green Flow Cytometry Assay Kit (catalog #: C10427; Thermo Fisher Scientific) following manufacturer's instructions.

### EdU NK Cell Turnover Assay

EdU was prepared as per manufacturer's instructions, and either 200 μg/200 μl/mouse or vehicle (DMSO) was injected i.p. on Day 0 and Day 2. Mice were then sacrificed at different timepoints. At each timepoint, spleen and BM from 1× femur were harvested from each mouse and made up as single cell suspension. Organs were lysed with RBC lysis buffer and stained for surface markers. EdU detection was followed exactly as per manufacturer's instructions (catalog #: C10632; Thermo Fisher Scientific) and analyzed on the FACS Verse.

### Total Cell Number and Mean Division Number Determination

The precursor cohort method used to determine mean division numbers has been published (51). Plotting the mean division number (MDN) of cell populations against time enables estimates and comparisons of division rates. MDN is the average number of divisions the initial cohort has undergone, where total cohort number describes the number of founding cells initially present within a population. To determine the MDN, each generation number *i* is multiplied by the fraction of the cohort that had undergone *i* divisions, and these values are summed for each generation:

$$\begin{array}{l} Mean\;division\;number=\sum \left(i\;\times \;\frac{{cohort\;number}_{i}}{total\;cohort\;number}\right)\; \end{array}$$

where i is division number.

### Plate Bound Assay

For the measurement of IFN-γ production, splenocytes were activated in 96 well plates with hIL-15 (50 ng/ml), IL-12 (10 ng/ml, Peprotech), IL-18 (100 ng/ml), PMA (10 ng/ml), Ionomycin (1 μg/ml), or coated with anti-NK1.1 (25 μg/ml, BioLegend), anti-NKp46 (10 μg/ml, BioLegend), or LY49H (10 μg/ml, BioLegend). Cells were incubated with monensin and brefeldin A (BD GolgiPlug and GolgiStop) in complete medium for 4 h at 37°C. The cells were subjected to surface staining and intracellular staining was performed by use of the FoxP3/Transcription Factor Staining Buffer Set (eBioscience).

### Tumor Cell Lines

MCA1956 fibrosarcoma cell line was derived from a B6.WT female mouse (gift of Robert Schreiber) and the SM1-LWT1 melanoma cell line was generously provided by Mark Smyth. Both MCA1956 and SM1-LWT1 melanoma cell lines were cultured in DMEM media containing 10% FCS (GE Healthcare Life Sciences), 100 U/mL penicillin (Sigma), 100 mg/mL streptomycin (sigma), 1 mM glutamax (Gibco) and sodium pyruvate (Gibco).

### Experimental Tumor Metastasis

Single-cell suspensions of 5 × 10^5^ MCA1956 cells were injected s.c. into the tail vein of the indicated strains of mice or s.c. Mice were sacrificed and lungs and/or tumors were harvested on day 14. Tumor weights were recorded and lungs were injected with Indian Ink, washed twice in PBS and fixed in Feketes solution overnight to count metastases.

### Sample Preparation, RNA Sequencing, and Bioinformatics Analysis

RNA isolation from sorted *ex vivo* NK cells was extracted using the RNeasy Plus Mini Kit (#74134, QIAGEN, Hilden, Germany), according to the manufacturer's instructions. Purified RNA was measured using an Agilent 2200 TapeStation System (Agilent) with High Sensitivity (HS) RNA ScreenTapes (#5067-5579, Agilent). Next-generation sequencing libraries were generated using 100 ng RNA from samples with distinct 18S and 28S peaks and RNA Integrity Number values ≥ 9, using the NEBNext Ultra II Directional RNA Library Prep Kit for Illumina (#E7760L, New England Biolabs) according to the manufacturer's instructions. Approximately 20 million reads per sample were obtained by pooling RNA libraries and performing single-end 75 bp sequencing. Sequencing was performed in the Genomics Laboratory at the Walter and Eliza Hall Institute on a NextSeq 500 next-generation sequencer (Illumina). Single-End reads 35–76 bp in length corresponding to *Cish*^−/−^ and WT *Cish*^+/+^ NK cells (three biological replicates per group) were quality checked using fastqc (52). Low quality bases and TrueSeq Adapters were trimmed using trimmomatic (53). Reads were aligned to 10 mm using STAR (54). The aligned reads were summarized at the gene-level using featureCounts (55). Genes were filtered from downstream analysis if they failed to achieve a CPM (counts per million mapped reads) value of at least 0.5 in at least three libraries. Counts were converted to log2-CPM, TMM normalized and precision weighted with the voom function of the limma package (56, 57). A linear model was fitted to each gene and empirical Bayes moderated t-statistics were used to assess differences in expression (58). Genes were called differentially expressed if they achieved a false discovery rate of 0.05 or less. Differential gene expression analysis, GO and KEGG pathway enrichment analysis were done using the R/Bioconductor package edgeR (59) and limma (57).

## Data Availability Statement

The raw data supporting the conclusions of this article will be made available by the authors, without undue reservation, to any qualified researcher.

## Ethics Statement

The animal study was reviewed and approved by The Walter and Eliza Hall Institute Animal Ethics Committee.

## Author Contributions

RD, WG, SH-Z, RH, JR, FS-F-G, and MD performed experiments. RD, FS-F-G, and NH designed experiments and analyzed the data. RD, GG, JN, FS-F-G, MD, and NH contributed intellectual input and helped to interpret data. NH led the research program. RD and NH wrote the manuscript.

### Conflict of Interest

JR and NH are founders and shareholders of oNKo-Innate Pty Ltd. NH receives research funding from Servier and Anaxis Pharma Pty Ltd. NH and FS-F-G have a funded research collaborative agreement with Paranta Biosciences Ltd. The remaining authors declare that the research was conducted in the absence of any commercial or financial relationships that could be construed as a potential conflict of interest.
