# Supplementary material for: NK Cell Priming From Endogenous Homeostatic Signals Is Modulated by CIS
Source: Front Immunol. 2020 Jan 31;11:75. doi: 10.3389/fimmu.2020.00075 (PMC7005222; doi:10.3389/fimmu.2020.00075)
Supplement: Supplementary file 1 [file Data_Sheet_1.PDF]

Supplemental Figure 1

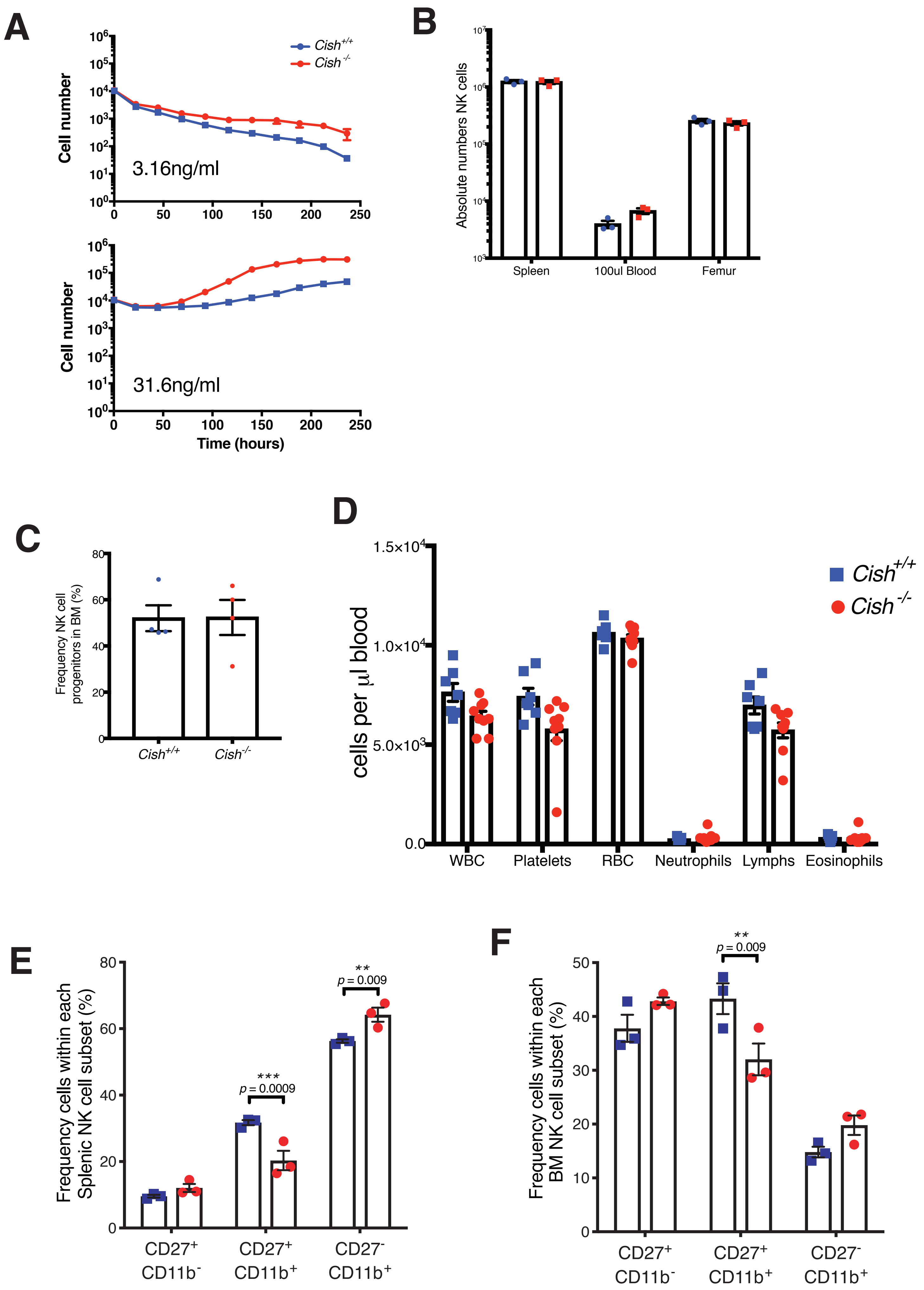

Supplemental Figure 1: (**A**) Purified and CTV-labelled *Cish*$^{-/-}$ and *Cish*$^{+/+}$ NK cells were seeded at 1x10^4 cells/well into round wells containing different concentrations of IL-15. Cells were incubated at 37°C in a humidified environment containing 5% CO2 for 240 hours. Total cell numbers over time are presented. **(B)** BM, spleen and blood NK cells from *Cish*$^{-/-}$ and *Cish*$^{+/+}$ mice were phenotypically analysed (CD3$^-$, CD19$^-$, NK1.1$^+$, NKp46$^+$) and enumerated by flow cytometry. (**C**) Femurs from 6-8 week-old *Cish*$^{+/+}$ and *Cish*$^{-/-}$ mice were flushed and NK cell progenitor (CD122$^+$IL7R$^+$NK1.1$^-$DX5$^-$CD3$^-$CD19$^-$) frequency quantified by flow cytometric analysis. **(D)** Cell composition of blood from *Cish*$^{+/+}$ *and Cish*$^{-/-}$ mice were analysed and quantified using the Advia 2120i. (**E, F**) NK cell maturation subsets were quantified by flow cytometric analysis of CD27 and CD11b in *Cish*$^{+/+}$ and *Cish*$^{-/-}$ mice. Frequency of CD27$^+$CD11b$^-$, CD27$^+$CD11b$^+$ and CD27$^-$CD11b$^-$ cells were measured within NK cell populations from the **(E)** spleen and **(F)** BM **p<0.01, ***p<0.001 (unpaired Student's *t*-test)**.** (**A**-**C**, $n \geq 3$ biological replicates mean ± s.e.m. of; **D**, n=9 biological replicates mean ± s.e.m; **E,F,** n=3 biological replicates mean ± s.e.m).

Supplemental Figure 2

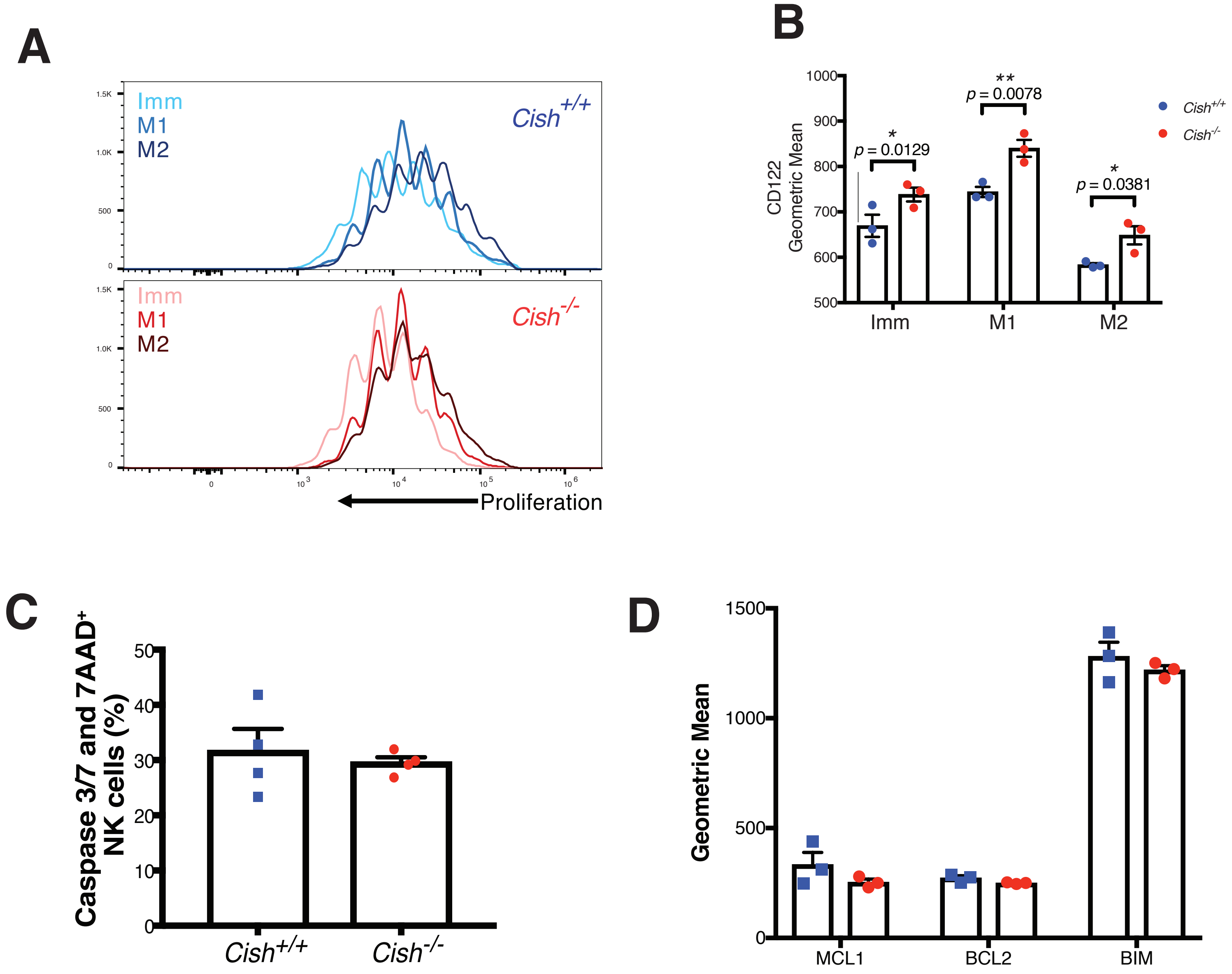

Supplemental Figure 2: (A) Flow cytometry was used to identify maturation subsets in splenic NK cells from 6-8 week-old *Cish*$^{+/+}$ and *Cish*$^{-/-}$ mice and Imm, M1 and M2 subsets were sorted, labelled with CFSE and cultured in NK cell media with 50ng/ml IL-15 for 7 days. Representative histograms of each subset are shown. **(B)** Flow cytometry was used to quantify the geometric mean of CD122 expression on *Cish*$^{+/+}$ and *Cish*$^{-/-}$ NK cells $*p<0.05$, $**p<0.01$ (unpaired Student's *t*-test). (**C**) Frequency of dying NK cells were quantified by phenotypic analysis of markers of apoptosis and necrosis using the CellEvent™ Caspase-3/7 Green Flow Cytometry Assay Kit. (**D**) Flow cytometry was used to quantify the geometric means of intracellular pro- and anti-apoptotic proteins. (**A**, one representative of two independent experiments; **B**, n=3 biological replicates mean ± s.e.m.; **C**, n=4 biological replicates mean ± s.e.m.; **D**, n=3 biological replicates mean ± s.e.m).

Supplemental Figure 3

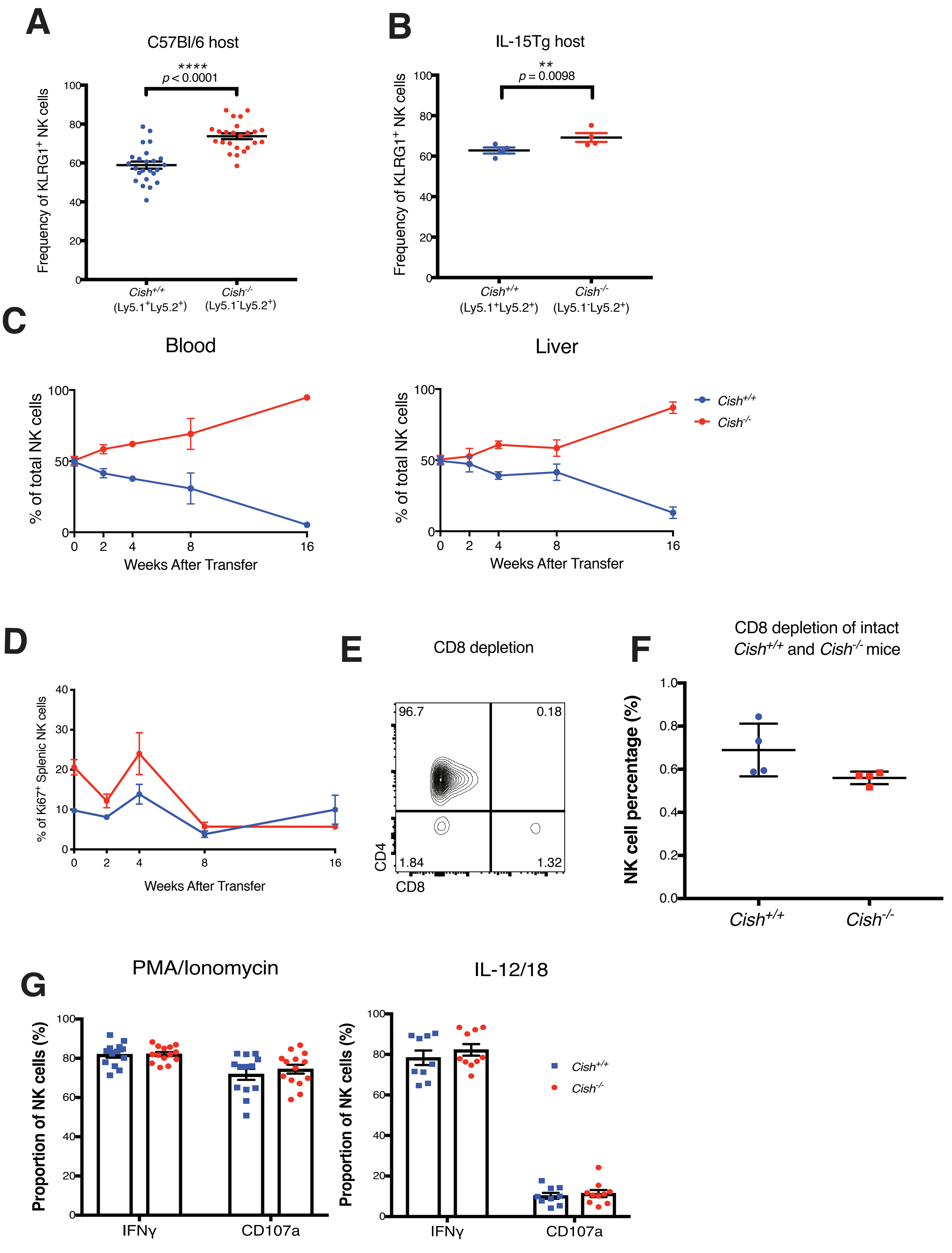

Supplemental Figure 3: (A, B) Haematopoietic chimeras were generated by injecting irradiated host mice (C57bl/6 or IL-15Tg) with equal amounts of control *Cish*$^{+/+}$ (Ly5.1^{+}Ly5.2^{+}) and *Cish*$^{-/-}$ (Ly5.2^{+}) bone marrow. After 6 weeks, NK1.1^{+}NKp46^{+}CD49b^{+} cells were analysed for expression of Ly5.1 and Ly5.2 to deduce donor origin and KLRG1 to measure maturation $^{**}p<0.01$, $^{****}p<0.0001$ (unpaired Student's *t*-test). (**C**) Adoptive transfer models were generated by injecting *Rag*2$^{-/-}\gamma_c^{-/-}$ mice with 1 x 10^5 FACS sorted *Cish*$^{+/+}$ (Ly5.1^{+}Ly5.2^{+}) and *Cish*$^{-/-}$ (Ly5.2^{+}) NK1.1^{+}NKp46^{+}CD49b^{+} NK cells. NK cells from the blood **(left panel)** and liver **(right panel**) were analysed for expression of Ly5.1 and Ly5.2 to deduce donor origin at indicated time points. **(D)** Adoptive transfer models were generated as above. Splenic NK cells were analysed for Ki67 expression at the indicated time points. (**E**) Reconstituted Rag2$^{-/-}\gamma_c^{-/-}$ mice were injected i.p. once per week for 4 weeks with anti-CD8 to deplete T cells and a representative plot of total CD8 T cell depletion is shown. (**F**) 6-8 week-old *Cish*$^{+/+}$ and *Cish*$^{-/-}$ mice were injected i.p. once per week for 4 weeks with anti-CD8 to deplete T cells. Splenic NK cell frequency is shown. (**G**) Total splenic cells were harvested from 6-8 week-old *Cish*$^{+/+}$ and *Cish*$^{-/-}$ mice and depleted of red blood cells. Cells were stimulated with PMA and Ionomycin (**left panel**) or IL-12 and IL-18 (**right panel**). Flow cytometric analysis of NK cells and their IFN-γ production and CD107a (LAMP-1) expression was measured after 4 hours. (**A**, **B**, mean ± s.e.m. of $n\geq3$ biological replicates; **C, D**, mean ± s.e.m. of $n=3$ biological replicates at each timepoint; **E**, representative FACS plot; **F**, mean ± s.e.m. of $n=4$ biological replicates;

G, mean ± s.e.m. of $n \geq 9$ biological replicates and is representative of 5 independent experiments.)
